# Metformin and feeding increase levels of the appetite-suppressing metabolite Lac-Phe in humans

**DOI:** 10.1038/s42255-024-01018-7

**Published:** 2024-03-18

**Authors:** Barry Scott, Emily A. Day, Katie L. O’Brien, John Scanlan, Grace Cromwell, Aine Ni Scannail, Marie E. McDonnell, David K. Finlay, Lydia Lynch

**Affiliations:** 1 School of Biochemistry and Immunology, Trinity Biomedical Sciences Institute, Trinity College Dublin, Dublin, Ireland; 2 Division of Endocrinology, Brigham and Women’s Hospital, Harvard Medical School, Boston, MA USA; 3 School of Pharmacy and Pharmaceutical Sciences, Trinity Biomedical Sciences Institute, Trinity College Dublin, Dublin, Ireland

**Keywords:** Metabolomics, Type 2 diabetes, Metabolism, Mechanism of action

## Abstract

Metformin, a widely used first-line treatment for type 2 diabetes (T2D), is known to reduce blood glucose levels and suppress appetite. Here we report a significant elevation of the appetite-suppressing metabolite *N*-lactoyl phenylalanine (Lac-Phe) in the blood of individuals treated with metformin across seven observational and interventional studies. Furthermore, Lac-Phe levels were found to rise in response to acute metformin administration and post-prandially in patients with T2D or in metabolically healthy volunteers.

## Abstract (web summary)

Metformin treatment was found to be associated with acute increases in the appetite-suppressing metabolite Lac-Phe in several human observational and interventional studies.

## Main

Metformin is prescribed to over 150 million people worldwide and acts to reduce blood glucose^1,2^ and weight^3,4^ in patients with type 2 diabetes (T2D). Despite its widespread use, the exact mechanisms underlying its therapeutic effects remain incompletely understood^2^. At higher concentrations, metformin can inhibit complex 1 of the electron transport chain^5^. However, there is uncertainty regarding whether the physiological concentration reached in the body is sufficient for this inhibition^5^. Certain tissues, particularly the intestines, may exhibit targeted inhibition of complex 1, reflecting a reported 30–300 times higher metformin concentration compared to the bloodstream^6^. Although other biguanide class drugs, such as phenformin, raised concerns over lactic acidosis^7^, the association of metformin with this condition is exceptionally rare. Lactate levels have been shown to increase in T2D cohorts with metformin use, but the effect is not universal (see ref. ^8^ for a review). It has been reported that lactate is increased in the portal vein, which drains from the gastrointestinal tract, in response to metformin^9,10^. The impact of metformin on weight loss is attributed to its appetite-suppressing properties, although the mechanisms involved are still unclear. Metformin-induced increases in the hormone GDF15 were proposed to mediate appetite suppression^11,12^, but recent findings have challenged whether this is the only or, indeed, the most important mechanism involved^13^.

*N*-lactoyl phenylalanine (Lac-Phe) is a metabolite generated by the peptidase carnosine dipeptidase 2 (CNDP2)^14^, which enzymatically fuses lactate and phenylalanine. Lac-Phe was recently shown to be an appetite suppressor when administered to obese mice and was correlated to weight loss in humans engaged in regular exercise^15^. In humans, Lac-Phe was also reported to increase with intense exercise^14,15^, in phenylketonuria^14^ and in mitochondrial disease (MELAS)^16^. However, whether Lac-Phe might contribute to the appetite-suppressing activities of metformin treatment has not been investigated.

**Fig. 1:**
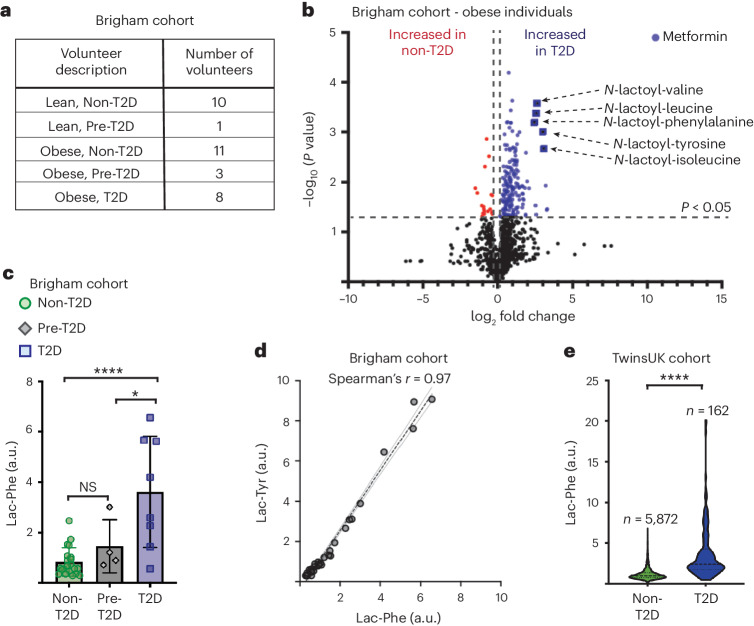
Elevated serum Lac-Phe in patients with T2D. **a**, Distribution of 33 non-diabetic (non-T2D), pre-diabetic (pre-T2D) and diabetic (T2D) participants in the Brigham and Women’s Hospital cohort study. **b**, Volcano plot comparing metabolomes of obese non-T2D (*n* = 11) and obese T2D (*n* = 8) individuals. Significantly upregulated (blue) and downregulated (red) metabolites are shown, and *N*-lactoyl amino acids are highlighted. **c**, Lac-Phe levels in non-T2D (*n* = 21), pre-T2D (*n* = 4) and T2D (*n* = 8) individuals (*P = 0.0198 and ****P < 0.0001; NS, non-significant). **d**, Spearmanʼs rank correlation between Lac-Phe and *N*-lactoyl-tyrosine (Lac-Tyr) in the total Brigham and Women’s Hospital cohort (*n* = 33). Graph shows mean linear regression with 95% confidence intervals. **e**, Lac-Phe in non-T2D (*n* = 5,876) and T2D (*n* = 162) individuals from the TwinsUK cohort (****P < 0.0001). Data are mean ± s.d. (**c**); violin plot with median (dashed line) plus maximum and minimum quartiles (dotted line) (**e**). Data were analysed using two-tailed Student’s *t*-test (**b**,**e**) or one-way ANOVA with Dunnett’s post test (**c**). Brigham cohort, Brigham and Women’s Hospital cohort. Source data

In the present study, serum samples were collected from 33 volunteers at Brigham and Women’s Hospital, comprising lean non-T2D, lean pre-T2D, obese non-T2D, obese pre-T2D and obese T2D volunteers (Fig. 1a). Untargeted metabolomic profiling was performed using ultra-high-performance liquid chromatography–tandem mass spectroscopy (UPLC–MS/MS), quantifying 1,168 serum metabolites. These data show a marked increase in all measured *N*-lactoyl amino acids in obese T2D volunteers compared to obese non-T2D volunteers (Fig. 1b). These increases were not related to the body mass index (BMI) of individuals but, rather, to T2D. Curiously, *N*-lactoyl amino acids are not commonly reported in metabolomic datasets; only three studies out of 2,621 in the online repository Metabolomics Workbench report Lac-Phe measurements. It transpires that *N*-lactoyl amino acids were mislabelled in metabolomic studies. For instance, a recent study detected increases in *N*-lactoyl amino acids in a subgroup of T2D volunteers, but *N*-lactoyl amino acids were erroneously reported as 1-carboxyethyl amino acids^17^. Other studies simply report Lac-Phe as unknown compound X-15497 (Extended Data Table 1).

**Fig. 2:**
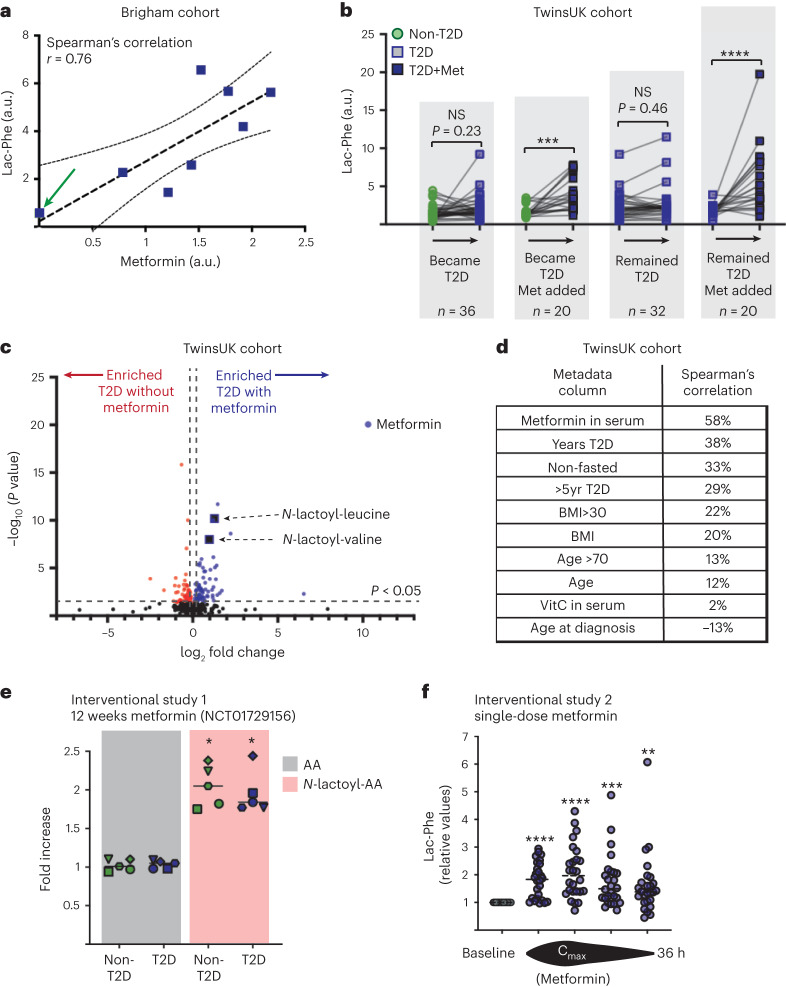
Metformin treatment increases serum Lac-Phe. **a**, Spearmanʼs rank correlation between metformin and Lac-Phe levels in obese T2D individuals (*n* = 8) for the Brigham and Women’s Hospital cohort. Graph shows mean linear regression with 95% confidence intervals. One volunteer (highlighted with an arrow) had stopped taking their metformin medication. **b**, TwinsUK volunteers provided three serum samples, with an average of 6.5 years between samples. Paired data are shown for volunteers whose T2D status changed and/or who commenced metformin treatment between samples (***P = 0.0002 and ****P < 0.0001; NS, non-significant). **c**, Volcano plot comparing metabolomes in T2D volunteers with (*n* = 71) and without (*n* = 91) metformin treatment. Metabolites significantly upregulated (blue) and downregulated (red) with metformin treatment are shown, and *N*-lactoyl amino acids and metformin are labelled. **d**, Spearmanʼs rank correlation in T2D samples (*n* = 162) versus non-metabolite metadata (Years T2D, years since T2D diagnosis; Non-fasted, volunteer had eaten within 6 h of sample collection; >5yr T2D, more than 5 years since T2D diagnosis; BMI, body mass index of volunteer; VitC in serum, ascorbate detected in metabolomics; Age at diagnosis, age when volunteer received a T2D diagnosis). **e**, Comparison of amino acid (AA) and *N*-lactoyl-amino acid (*N*-lactoyl-AA) levels after a 12-week metformin intervention, relative to pre-treatment levels, in a 2019 Danish study (NCT01729156). Fold increases for patients with recent-onset T2D and age/BMI-matched non-T2D controls (*n* = 12 per group) showing five separate AAs and the corresponding *N*-lactoyl-AA; phenylalanine (circle), tyrosine (square), valine (hexagon), leucine (triangle) and isoleucine (diamond) (*P < 0.05). **f**, Lac-Phe levels (relative to baseline) over 36 h after a single oral dose of metformin in a study involving 26 young healthy male volunteers. Lac-Phe values measured before the maximum metformin concentration (Cmax) in the serum, at the Cmax and after the Cmax. A final sample was taken 36 h after dosing (**P = 0.0086, ***P = 0.0005 and ****P < 0.0001). Data are individual data points (**a**,**f**), individual paired data points (**b**) and mean values (**e**). Data were analysed using two-way ANOVA with Fisher’s LSD post test (**b**), two-tailed Student’s *t*-test (**c**), Welch’s two-sided Student’s *t*-test (**e**) and one-sample *t*-test against a theoretical value of 1 (**f**). Brigham cohort, Brigham and Women’s Hospital cohort. Source data

**Extended Data Fig. 1:**
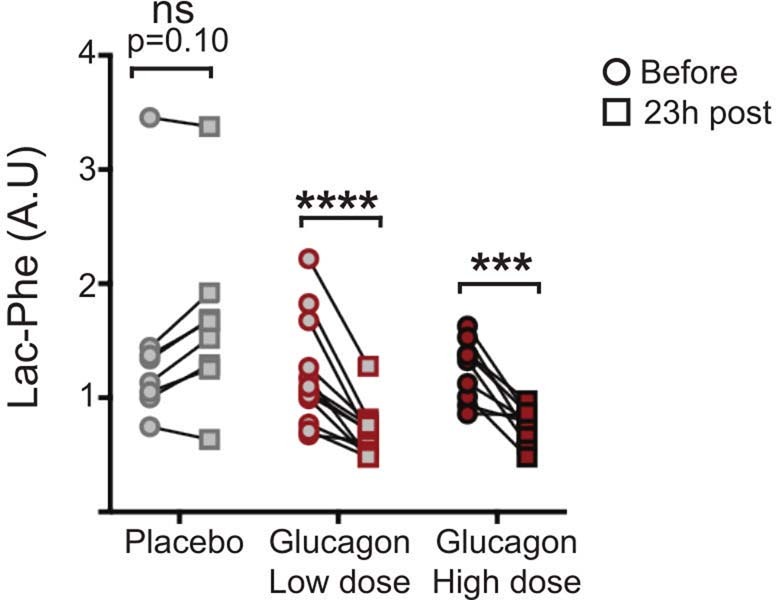
Glucagon does not induce Lac-Phe levels. LacPhe levels before and 23 hours post treatment with low (GCG 12.5 ng/kg/min, n = 12) or high (GCG 25 ng/kg/min, n = 9) dose glucagon or placebo control (n = 8). Data shown is individual paired data points for Lac-Phe pre and post treatment. Data was analysed using a 2-way ANOVA with a Sidak post-test. (***, p = 0.008). Source data

Given the interesting properties of Lac-Phe as an appetite suppressor^15^, we focused on this particular *N*-lactoyl amino acid. These data show that Lac-Phe levels were 5.7-fold higher in obese T2D individuals compared to obese non-T2D control volunteers (Fig. 1c). A significant increase was also observed between obese T2D and pre-diabetic individuals (Fig. 1c). Lac-Phe concentrations tightly correlated with levels of other *N*-lactoyl amino acids (Fig. 1d). To support these findings, the serum metabolomic data from the extensive TwinsUK cohort were investigated^18^. This dataset, consisting predominantly of middle-aged female participants, provided over 2,000 individual metabolic profiles with over 6,000 serum samples collected from 1997 to 2012 (Extended Data Fig. 1). In this cohort, Lac-Phe levels were also increased in volunteers with T2D (Fig. 1e). Considering the prevalence of metformin use in individuals with T2D, we considered whether the serum levels of Lac-Phe were linked with metformin treatment. Interestingly, there was a strong correlation between Lac-Phe and metformin concentrations (Spearmanʼs ρ = 0.76) among individuals with T2D in the Brigham and Women’s Hospital cohort (Fig. 2a). Although metformin use was an inclusion criterion for the T2D volunteers in this particular study, in one volunteer’s sample metformin was not detected, and this coincided with the lowest observed Lac-Phe measurement (Fig. 2a). It emerged that this volunteer had actually discontinued taking metformin. These data led us to hypothesize that the increased Lac-Phe in the serum of individuals with T2D might be driven by metformin rather than T2D itself.

The TwinsUK dataset offered a unique opportunity to test this hypothesis, as it could be used to observe metabolic changes over time. Each volunteer had provided three serum samples between 1997 and 2012, with an average of 6.5 years between blood draws. This was a period where metformin’s role in T2D management expanded considerably. These data can be used to trace the trajectory of Lac-Phe levels in volunteers whose T2D and metformin status changed over time (Fig. 2b). Volunteers newly diagnosed with T2D and starting metformin treatment exhibited a significant elevation in Lac-Phe levels. In contrast, those newly diagnosed with T2D but not initiating metformin therapy did not show a significant change in Lac-Phe levels (Fig. 2b). Similarly, among volunteers consistently diagnosed with T2D over successive blood draws, the introduction of metformin treatment led to a substantial rise in Lac-Phe levels. In contrast, volunteers who remained with T2D but did not initiate metformin therapy did not show a significant change in Lac-Phe levels (Fig. 2b). Indeed, comparative analysis of individuals with T2D who were either taking metformin or not on this medication revealed that among the most significantly increased metabolites were the two *N*-lactoyl amino acids, Lac-Phe and *N*-lactoyl-valine (Fig. 2c). When the metadata of T2D volunteers were analysed to see what factors best correlated to Lac-Phe levels, we discovered that metformin treatment was the most significant factor, ahead of ‘years since T2D diagnosis’ and ‘BMI score’ (Fig. 2d). This provided further evidence that starting metformin treatment leads to higher serum Lac-Phe levels.

To establish a causal link between metformin treatment and elevated Lac-Phe levels, data from two previously published interventional studies were analysed. In a 2019 Danish study^19^ (NCT01729156), patients with recent-onset T2D who were over 50 years of age and BMI-matched non-diabetic controls, with 12 volunteers in each group, underwent 12 weeks of metformin treatment. Serum metabolomics was performed before and after the 12-week intervention. Although no difference was observed in the levels of phenylalanine, tyrosine, valine, leucine and isoleucine in the serum of either T2D or non-T2D groups, there were clear and significant increases in the corresponding *N*-lactoyl amino acids, including Lac-Phe (Fig. 2e). Post-treatment untargeted metabolomic analysis measured a more than 80% increase in Lac-Phe levels in both groups (Fig. 2e). Indeed, even acute metformin treatment leads to increased Lac-Phe levels. A Jordanian study^20^ gave 26 young healthy men a single oral dose of metformin and performed non-targeted metabolomic analysis over the course of 36 h. Levels of Lac-Phe quickly increased alongside those of metformin, with a peak in serum Lac-Phe observed corresponding to the maximum concentration observed (Cmax) of metformin (Fig. 2f). Taken together, these interventional studies that encompassed both long-term and acute metformin dosing provide compelling evidence that metformin treatment induces elevated serum Lac-Phe levels.

It would be interesting to investigate the impact of other appetite-suppressing T2D drugs on Lac-Phe levels, focusing on GLP-1 receptor agonists, dual GIP-GLP-1 receptor agonists and triple glucagon-GIP-GLP-1 agonists in development^21^. Although suitable metabolomics datasets on GLP-1 and dual GIP-GLP-1 receptor agonists were not available to us, we did analyse a cohort study^22^ where glucagon was administered to healthy volunteers. In contrast to the increase in Lac-Phe levels seen with metformin, glucagon administration resulted in reduced Lac-Phe (Extended Data Fig. 1). This observation aligns with glucagon’s known role in stimulating gluconeogenesis, for which lactate is a key substrate.

**Fig. 3:**
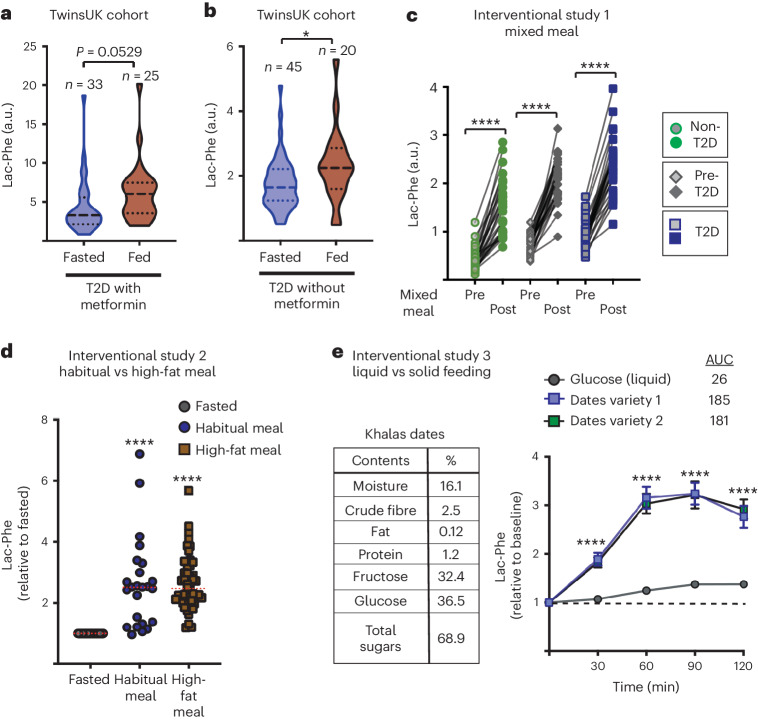
Feeding increases serum Lac-Phe concentrations. **a**,**b**, Lac-Phe levels in T2D volunteers with (**a**) or without (**b**) metformin treatment (sample sizes indicated) and under fasted (blue bars) and non-fasted (red bars) conditions, where volunteers had eaten within 6 h (*P = 0.034). **c**, Lac-Phe levels 30 min before and 1 h after a standardized MMT involving non-T2D (*n* = 10), pre-T2D (*n* = 10) and T2D (*n* = 10) volunteers. Each volunteer completed the test on three separate days, resulting in a total of 90 paired samples (*n* = 30 for each group) (****P < 0.0001). **d**, Lac-Phe levels after a volunteer’s habitual meal (*n* = 24) or high-fat meal (*n* = 48), relative to fasted levels, in 24 overweight/obese sedentary men. Serum samples were taken before breakfast and approximately 45 min after dinner (****P < 0.0001). **e**, Lac-Phe levels after feeding with liquid glucose or dates. Left, Khalas date nutritional information is given for reference. Twenty-one participants undertook three separate dietary challenges, consuming either a glucose solution (OGTT, *n* = 21) or ten fruits of the Deglet Nour (*n* = 21) or Khalas (*n* = 20, one volunteer did not participate) date varieties. Blood samples were collected before intake and at intervals up to 120 min afterward. Lac-Phe levels were normalized to fasting levels. The AUC is shown. Data are violin plots with median (dashed line) plus maximum and minimum quartiles (dotted line) (**a**,**b**), individual paired data points (**c**), mean (dotted line) and individual data points (**d**) and mean ± s.e.m. (**e**). Data were analysed using two-sided Studentʼs *t*-test (**a**,**b**), two-way ANOVA with Sidakʼs post test (**c**) or Tukeyʼs post test (**e**) or one-sample *t*-test against a theoretical value of 1 (**d**). Source data

**Extended Data Fig. 2:**
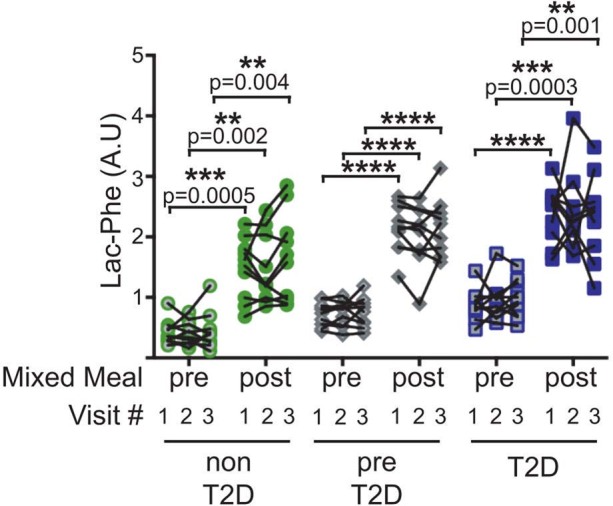
Stable baseline Lac-Phe levels in non-T2D, pre-T2D and T2D individuals. LacPhe levels 30 minutes before and 1 hour after a standardised mixed meal test involving non-T2D (n = 10), pre-T2D (n = 10), and T2D volunteers (n = 10). Each volunteer completed the test on 3 separate days (labelled visit 1, 2 and 3). (****, p < 0.0001). Data is individual linked measurements for each volunteer across the 3 visits. Data was analysed using a two-way ANOVA with a Tukey post-test comparing paired pre and post measurements for each group on each visit. Source data

When TwinsUK T2D volunteer metadata were analysed, we also noticed that there was a high correlation between Lac-Phe levels and being ‘Non-Fasted’ (Fig. 2d). It is well established that lactate levels increase after eating, and so we were intrigued to investigate whether the highly elevated Lac-Phe levels in metformin-treated T2D individuals were impacted by the feeding or fasting state. In the TwinsUK cohort, although most samples were collected under fasted condition, there was a subset of T2D samples from individuals who had eaten within 6 h of sample collection. When the data for fed and fasted metformin-treated T2D individuals were interrogated, a trend toward increased Lac-Phe levels was observed in the fed volunteers (Fig. 3a). Although, overall, the Lac-Phe levels were lower in volunteers who were not taking metformin, significantly higher Lac-Phe was observed in fed rather than fasted volunteers, arguing that serum Lac-Phe levels are controlled by food intake (Fig. 3b). Given Lac-Phe’s appetite-suppressing properties, we speculated that its elevation post-meal could act as a feedback mechanism in appetite regulation. Analysis of data from a 2020 interventional study further substantiated these findings^23^. The study involved T2D, pre-diabetic and metabolically healthy volunteers, with ten individuals per group. For the T2D group participants who were on metformin, the medication was paused the night before each of the study days. On three separate days within a 1-month period, each group underwent a mixed-meal test—a liquid meal replacement shake plus a solid protein bar—resulting in 90 instances where metabolite levels were assessed before and after feeding. Lac-Phe stood out as one of the metabolites with the most significant response to food intake. In all 90 cases, a post-prandial increase in Lac-Phe levels was observed, with an average increase of 186% 1 h after meal consumption (Fig. 3c). Given that volunteers were sampled on three separate occasions within a single month, this provided a unique opportunity to assess inter-individual differences in Lac-Phe levels. We examined the consistency of individual Lac-Phe levels across these three sampling points. Individuals had consistent baseline Lac-Phe and post-prandial Lac-Phe across these three visits (Extended Data Fig. 2). The variation between the three visits by each individual accounted for only 0.08% of the total variation in the study (Extended Data Fig. 2). Multiple factors could contribute to inter-individual differences in baseline Lac-Phe levels. Such observations suggest the existence of distinct Lac-Phe setpoints among individuals. A 2021 Australian interventional cohort study^24^ provided additional evidence of Lac-Phe’s response to varying dietary challenges. This study involved 24 overweight/obese sedentary men who underwent assessments on different days under distinct dietary regimes. Serum samples were collected for metabolomic analysis before breakfast and after dinner, where the dinner was either their standard habitual diet or a high-fat diet. The results were striking, showing a greater than 160% increase in Lac-Phe levels both in participants who consumed their standard meal and in those consuming high-fat meals (Fig. 3c). These data suggest that Lac-Phe levels increase with diverse types of diet. Notably, in all cases above, the volunteers consumed a solid component in their meal. To test whether a liquid diet also induced Lac-Phe levels, the data were analysed from volunteers before and after consuming either liquid sugar (glucose) or sugar-rich date fruits. These data were from a 2018 nutritional challenge study conducted in Qatar^25^. In this study, 21 healthy volunteers underwent plasma metabolomic assessments after the consumption of either Khalas or Deglet Nour date fruits or, alternatively, oral glucose. Notably, the composition of dates is predominantly sugars with minimal protein content and a small amount of fibre (Fig. 3d). Each volunteer took part in three separate dietary challenges, spaced a week apart, involving the consumption of either ten date fruits or a glucose drink. Blood samples were collected at baseline and at intervals of 30 min, 60 min, 90 min and 120 min after intake. Lac-Phe levels increased over 220% in response to both date fruit varieties (Fig. 3e). In contrast, the response to the liquid glucose challenge, although significant, was considerably lower, with only a 37% increase in Lac-Phe levels measured. This differential response suggests that Lac-Phe’s regulatory role in appetite might vary between solid and liquid meal intakes, potentially offering new insights into how different food forms impact hunger signalling. Our analysis conclusively demonstrates that Lac-Phe levels consistently rise after meals, a finding evident in both observational and interventional studies.

In conclusion, our study demonstrates that metformin, a well-tolerated drug with an excellent safety record, increases Lac-Phe levels. Another paper published in parallel with this work^26^ shows that Lac-Phe is increased in both mice and humans upon metformin treatment. The authors experimentally demonstrate, using CNDP2 knockout mice, that metformin inhibits complex 1 in the intestines, causing increased Lac-Phe levels, which contributes to metformin’s appetite-suppressing role. Our present work confirms and extends their results, demonstrating that Lac-Phe increases with a single dose of metformin. We clearly demonstrate that Lac-Phe increases are specific to metformin treatment rather than T2D status. In addition, we demonstrate that Lac-Phe increases in response to metformin treatment in both healthy individuals and those with T2D. Interestingly, our findings extend beyond Lac-Phe, showing that metformin increases levels of at least five *N*-lactoyl amino acids, thus broadening understanding of metformin’s metabolic effects. Another novel finding was that Lac-Phe levels increase post-prandially, paralleling the regulatory patterns of other appetite suppressants, such as ghrelin and leptin, and providing a rationale for the role of Lac-Phe in regulating appetite. This suggests that deliberate targeting of Lac-Phe pharmacologically could lead to stronger appetite-suppressing effects and result in a new class of safe and effective drugs to treat obesity.

## Methods

### Brigham and Women’s Hospital cohort

The present study was conducted at Brigham and Women’s Hospital from August 2019 to December 2019. The participants were categorized into five groups: obese with T2D, obese pre-diabetic, obese non-T2D, lean pre-diabetic and lean non-T2D.

All patient procedures took place at Brigham and Women’s Hospital. In total, 33 volunteers were recruited for the study. The breakdown of the groups is as follows:

Obese T2D: eight participants (three women, five men) with a BMI range of 30–45 kg m^−2^ and hoemoglobin A1c (HbA1c) levels between 5.7% and 9%. It is important to note that one participant was not adhering to their prescribed metformin regimen. Another individual had a BMI marginally below the initial target (29.8 kg m^−2^). Obese pre-diabetic: three participants (two men, one woman), including one individual with a BMI slightly below the initial target (28.3 kg m^−2^). Obese non-T2D: 11 participants (three men, eight women), with BMI ranges primarily within 30–45 kg m^−2^, including one individual with a BMI slightly below the initial target (29 kg m^−2^). Lean pre-diabetic: one participant (one man) with a BMI of 19.6 kg m^−2^. Lean non-T2D: ten participants (two men, eight women), all within the targeted BMI range for lean controls.

### Ethics declarations

Patients attending the diabetes clinic at Brigham and Women’s Hospital were recruited prospectively. All patients provided written informed consent. This study was reviewed and approved by the Brigham and Women’s Hospital institutional review board (IRB 2020P002554 and 2019P001128).

### Recruitment methodology

Volunteers for this study were recruited from the diabetes clinic and/or the weight management clinic at Brigham and Women’s Hospital for the obese groups with or without T2D. In addition, flyers placed in clinical and non-clinical areas within the hospital were used to recruit lean control subjects.

The study included men and women aged 18–75 years. For lean controls, Caucasian and African American individuals were required to have a BMI of 18.5–24.9 kg m^−2^, whereas Asian adults needed a BMI of 18.5–23.0 kg m^−2^. The obese cohorts, whether with or without pre-diabetes or diabetes, had inclusion criteria specifying a BMI of 30–45 kg m^−2^ and HbA1c levels between 5.7% and 9.5%. Individuals diagnosed with diabetes were primarily managed with metformin, with some also receiving insulin. However, the study excluded current smokers; individuals who were pregnant or planning pregnancy during the study; individuals with serious uncontrolled cardiovascular, nervous system, pulmonary, renal or gastrointestinal diseases; individuals with serologic evidence of HIV, hepatitis B or hepatitis C; individuals with a history of tuberculosis; individuals with liver disease characterized by elevated hepatic enzymes; or individuals with any hematologic abnormalities within the last 12 months, including a white blood count outside the range of 3,000–14,000 cells per microlitre, lymphocyte count below 500 cells per microlitre, platelet count below 150,000 cells per microlitre, hoemoglobin levels less than 10 g dl^−1^ or neutrophil count below 2,000 cells per microlitre. Additional exclusion criteria were poor glycaemic control (HbA1c over 9.5% for individuals in the diabetes group), major psychiatric illness or ongoing alcohol or drug abuse.

### TwinsUK cohort overview

The TwinsUK study involved 2,069 participants, predominantly of European ancestry, from the TwinsUK registry^18^. The cohort was primarily female (96.6%), with participants aged between 32 years and 73 years at the first of three visits, which spanned 8–18 years. Throughout the study, a total of 6,196 samples were collected. Among these, 86 volunteers were identified as having T2D in at least one blood draw, resulting in 162 T2D samples. Notably, metformin was detected in 71 of these T2D samples. Detailed methodologies and further insights for this dataset were previously described^27^.

### Danish metformin intervention study overview

The 2019 Danish study at Aarhus University Hospital involved a 12-week treatment of metformin for patients with recent-onset T2D and age/BMI-matched non-diabetic controls. This intervention included 24 individuals with T2D and 12 non-diabetic controls, all receiving 2,000 mg of metformin daily. For a comprehensive understanding of the study design, participant demographics and methodologies, see the original publication^19^.

### Jordanian/Saudi acute metformin study overview

Conducted at the Jordan Center for Pharmaceutical Research in Amman, Jordan, the 2021 study involved 26 healthy young male subjects from Jordan. Each participant, aged 18–50 years, received a single oral dose of 500-mg metformin hydrochloride. Blood samples were collected at five specific timepoints: before drug administration, 1.5 h before the maximum serum concentration of metformin (Cmax), at Cmax, 2 h after Cmax and 36 h after administration, amounting to a total of 130 samples for metabolomic analysis. For detailed methodologies, see the original publication^20^.

### One-hour post-mixed-meal metabolic response study

In the 2020 study conducted at Nuvisan GmbH in Neu-Ulm, Germany, 30 participants were divided into three groups: T2D, pre-diabetic and metabolically healthy, each comprising ten individuals. The research centred around a standardized mixed-meal test (MMT), performed on three separate days, resulting in a total of 90 instances where metabolite levels were assessed before and after meal consumption. Serum samples were collected 30 min before and 1 h after the meal. Recruitment criteria included HbA1c, fasting and oral glucose tolerance test (OGTT)-challenged glucose, C-peptide and intact proinsulin measurements. For T2D participants on metformin, the medication was paused the evening before the MMT. Detailed methodology of the study can be found in the original publication^23^.

### Date fruit and OGTT study

Conducted at the Medical Laboratories Clinic in Doha, Qatar, the 2018 nutritional challenge study assessed the plasma metabolome in 21 healthy volunteers, comprising 13 females and eight males, after the consumption of Khalas and Deglet Nour date fruits compared to an OGTT. Volunteers participated in three separate challenges with a week’s interval between each, consuming either a glucose drink or ten fruits from the specified date varieties. Blood samples were taken at baseline and at 30 min, 60 min, 90 min and 120 min after intake. The study identified 1,089 blood circulating metabolites. Detailed methodology of the study can be found in the original publication^25^.

### High-fat diet intervention study

Conducted at the Australian Catholic University in 2018, a cohort study was conducted involving 24 overweight/obese sedentary men. Assessments were conducted on three separate days: initially under volunteers’ habitual diets, after a 5-day high-fat diet and after an additional 5 days of the high-fat diet with an exercise intervention. Participants were randomized into three groups to compare the effects of morning and evening exercise against no exercise. On the days of metabolomic sampling, participants refrained from exercise. Serum for metabolomics was drawn before breakfast at approximately 7:15 and again at 19:15, after an 18:30 dinner. Further details are available in the original publication^24^.

### Glucagon infusion metabolic impact study

This 2021 study^22^, a randomized, placebo-controlled trial conducted at the Translational Research Institute at AdventHealth in Orlando, Florida, USA, involved 33 healthy volunteers who were overweight or obese and who underwent a 72-h continuous glucagon or placebo infusion. The participants were allocated into three groups: a placebo group (*n* = 10), a low-dose glucagon group (GCG 12.5 ng kg^−1^ min^−1^, *n* = 12) and a high-dose glucagon group (GCG 25 ng kg^−1^ min^−1^, *n* = 11). Participants underwent five overnight stays, with the glucagon or saline infusions beginning after baseline assessments. Our metabolomics analysis was based on the blood samples drawn a half hour before and 23 h into the glucagon or placebo infusion, both times under fasting conditions. Due to missing metabolomics samples—at least one sample was absent for two participants in the placebo group and two in the high-dose glucagon group—the effective sample sizes for our analysis were adjusted to *n* = 8 for the placebo group, remained *n* = 12 for the low-dose glucagon group and were reduced to *n* = 9 for the high-dose glucagon group. For comprehensive methodology, see the original publications^22,28^ and ClinicalTrials.gov (NCT03139305).

### Untargeted global metabolite profiling

Metabolon, Inc. performed untargeted metabolite profiling for seven out of the eight cohorts involved in our comprehensive analysis. The Jordanian/Saudi acute metformin study is the only cohort for which Metabolon did not conduct the metabolomics analysis. Detailed methodologies for all cohorts can be found in their respective original publications.

### Example methods for Brigham and Women’s Hospital cohort

Sample preparation and analysis were carried out as described previously^29^ at Metabolon, Inc. In brief, sample preparation involved protein precipitation and removal with methanol, shaking and centrifugation. The resulting extracts were profiled on an accurate mass global metabolomics platform consisting of multiple arms differing by chromatography methods and mass spectrometry ionization modes to achieve broad coverage of compounds differing by physiochemical properties, such as mass, charge, chromatographic separation and ionization behaviour. The details of this platform were described previously^30^. Metabolites were identified by automated comparison of the ion features in the experimental samples to a reference library of chemical standard entries that included retention time, molecular weight (*m*/*z*), preferred adducts and in-source fragments, as well as associated mass spectrometry spectra, and were curated by visual inspection for quality control using proprietary software developed at Metabolon^29,31^.

### *N*-lactoyl amino acid identification

*N*-lactoyl amino acids have been labelled as unknown and mislabelled in existing metabolomic datasets. We systematically identified and corrected these mislabellings based on our updated mapping table, ensuring accurate representation of *N*-lactoyl amino acids in our study (Extended Data Table 1).

### Statistical methodology

Unless otherwise stated, untargeted metabolomics values were median normalized on a per-compound basis; for more details, see ref. ^29^. For all timecourse analyses across cohorts, Lac-Phe levels were normalized to individual baseline measurements to ensure consistent assessment of changes over time. Statistical significance in the volcano plots was determined using two-tailed Student’s *t*-test. For visualization, *P* values and fold changes were log transformed to −log_10_ and log_2_, respectively. For the Brigham and Women’s Hospital cohort, Lac-Phe levels were analysed using ordinary one-way ANOVA with Dunnett’s multiple comparisons test. Relationships between metformin levels and Lac-Phe in the Brigham and Women’s Hospital cohort were assessed using simple linear regression, with the goodness of fit expressed as an R^2^ value. In the TwinsUK cohort violin plot, differences in Lac-Phe distributions between non-T2D and T2D groups were evaluated using an unpaired *t*-test. Correlations were evaluated using Spearmanʼs rank correlation coefficients unless otherwise stated. To analyse the effect of T2D diagnosis and metformin initiation in the TwinsUK cohort, two-way ANOVA was conducted with multiple comparisons adjusted using Fisher’s least significant difference (LSD) test. To analyse the effect of fasted state on T2D in the TwinsUK cohort, unpaired *t*-tests were used. In the single-dose metformin study, one-sample *t*-tests were used to independently compare normalized Lac-Phe levels at each timepoint against the pre-metformin baseline. In the original publication for the Danish study (NCT01729156), Welch’s two-sample *t*-test on log-transformed data was used to assess statistical significance change from baseline sample to samples after 12 weeks. A mixed-effects model with Sidak’s multiple comparisons test was used to assess significance versus baseline in the mixed-meal intervention cohort. One-sample *t*-test was used to assess significance versus baseline in the high-fat diet cohort study. GraphPad Prism was used to calculate area under the curve (AUC) to compare date fruit interventions to an OGTT.

The quoted size increase in Lac-Phe for the four metformin cohorts was calculated as follows: for the Brigham and Women’s Hospital cohort, by dividing the average Lac-Phe levels in obese T2D by the average levels in obese non-T2D; in the TwinsUK cohort, by dividing the average Lac-Phe levels of T2D on metformin by the average levels of T2D not on metformin; for the Danish study (NCT01729156), by comparing average levels after a 12-week intervention versus before intervention for healthy controls and individuals with T2D. The quoted size increase in Lac-Phe for the fed cohort was calculated by dividing the average post-meal Lac-Phe values by the pre-meal values; notably, mixed-meal values were not normalized to baseline, unlike the other cohorts where baseline-normalized values were used.

### Software

Data and statistical analysis was performed using Python version 3.8 and the accompanying pandas 1.4.3 package. GraphPad Prism version 10.1.2 was also used.

### Reporting summary

Further information on research design is available in the Nature Portfolio Reporting Summary linked to this article.

### Supplementary information

Reporting Summary

Supplementary Data Metabolomics from Brigham and Women’s Hospital cohort and volunteer metadata.

### Source data

Source Data Fig. 1 Statistical source data.

Source Data Fig. 2 Statistical source data.

Source Data Fig. 3 Statistical source data.

Source Data Extended Data Fig. 1 Statistical source data.

Source Data Extended Data Fig. 2 Statistical source data.

## Figures and Tables

**Extended Data Table 1:**
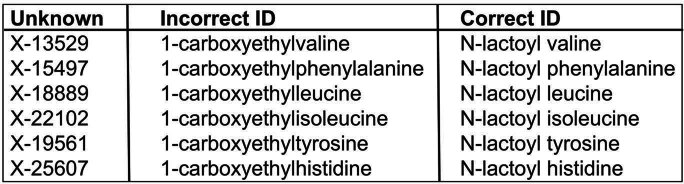
Correction of metabolite mislabelings

This table delineates the original unknown labels assigned to *N*-lactoyl amino acids (Unknown column), their previous misidentifications (Incorrect ID column) and their verified correct identifications (Correct ID column). These mappings were used to accurately identify metabolites reported in our cohorts.

## Data Availability

Metabolomic data for the Brigham and Women’s Hospital cohort are provided in the supplementary materials. Upon Access to the TwinsUK metabolomics dataset can be applied for through the TwinsUK Resource Executive Committee. We used data posted with the original manuscripts from the following three articles: Short-term variability of the human serum metabolome depending on nutritional and metabolic health status^23^; Metabolic changes of the blood metabolome after a date fruit challenge^25^; and Metformin increases endogenous glucose production in non-diabetic individuals and individuals with recent-onset type 2 diabetes^19^. Data from the following two articles can be requested from the corresponding authors: A metabolic pattern in healthy subjects given a single dose of metformin: a metabolomics approach^20^; and The effect of morning vs evening exercise training on glycaemic control and serum metabolites in overweight/obese men: a randomised trial^24^. Source data are provided with this paper.

## Code availability

Scripts for analysing data from the three publicly available cohorts are posted on GitHub at https://github.com/barryfscott/Metformin-Feeding-Lac-Phe-Analysis. For code related to non-public datasets, contact the corresponding author.
