# Supplementary material for: Metformin and feeding increase levels of the appetite-suppressing metabolite Lac-Phe in humans
Source: Nat Metab. 2024 Mar 18;6(4):651–8. doi: 10.1038/s42255-024-01018-7 (PMC11052712; doi:10.1038/s42255-024-01018-7)
Supplement: Supplementary file 1 — Reporting Summary [file 42255_2024_1018_MOESM1_ESM.pdf]

nature portfolio

Corresponding author(s): Lydia Lynch
Last updated by author(s): Feb 14, 2024

Reporting Summary

Nature Portfolio wishes to improve the reproducibility of the work that we publish. This form provides structure for consistency and transparency in reporting. For further information on Nature Portfolio policies, see our Editorial Policies and the Editorial Policy Checklist.

Statistics

For all statistical analyses, confirm that the following items are present in the figure legend, table legend, main text, or Methods section.

n/a	Confirmed	
☐	☒	The exact sample size (*n*) for each experimental group/condition, given as a discrete number and unit of measurement
☐	☒	A statement on whether measurements were taken from distinct samples or whether the same sample was measured repeatedly
☐	☒	The statistical test(s) used AND whether they are one- or two-sided<br>*Only common tests should be described solely by name; describe more complex techniques in the Methods section.*
☐	☒	A description of all covariates tested
☐	☒	A description of any assumptions or corrections, such as tests of normality and adjustment for multiple comparisons
☐	☒	A full description of the statistical parameters including central tendency (e.g. means) or other basic estimates (e.g. regression coefficient) AND variation (e.g. standard deviation) or associated estimates of uncertainty (e.g. confidence intervals)
☐	☒	For null hypothesis testing, the test statistic (e.g. *F*, *t*, *r*) with confidence intervals, effect sizes, degrees of freedom and *P* value noted<br>*Give P values as exact values whenever suitable.*
☒	☐	For Bayesian analysis, information on the choice of priors and Markov chain Monte Carlo settings
☒	☐	For hierarchical and complex designs, identification of the appropriate level for tests and full reporting of outcomes
☐	☒	Estimates of effect sizes (e.g. Cohen's *d*, Pearson's *r*), indicating how they were calculated

Our web collection on statistics for biologists contains articles on many of the points above.

Software and code

Policy information about availability of computer code

Data collection	No specific software was used to collect data.
Data analysis	Data analysis was performed using Python v3.8 and accompanying package pandas 1.4.3. GraphPad Prism v10.1.2 was also used

For manuscripts utilizing custom algorithms or software that are central to the research but not yet described in published literature, software must be made available to editors and reviewers. We strongly encourage code deposition in a community repository (e.g. GitHub). See the Nature Portfolio guidelines for submitting code & software for further information.

Data

Policy information about availability of data

All manuscripts must include a data availability statement. This statement should provide the following information, where applicable:

- Accession codes, unique identifiers, or web links for publicly available datasets
- A description of any restrictions on data availability
- For clinical datasets or third party data, please ensure that the statement adheres to our policy

Metabolomic data for the Brigham cohort are provided in the supplementary materials. Access to the TwinsUK metabolomics dataset can be applied for through the TwinsUK Resource Executive Committee.
We used data posted with the original manuscripts:from the following 3 studies:
Short-term variability of the human serum metabolome depending on nutritional and metabolic health status

Metabolic changes of the blood metabolome after a date fruit challenge.
Metformin increases endogenous glucose production in non-diabetic individuals and individuals with recent-onset type 2 diabetes

Data from the following 2 articles can be requested from the corresponding authors:
A Metabolic Pattern in Healthy Subjects Given a Single Dose of Metformin: A Metabolomics Approach
The effect of morning vs evening exercise training on glycaemic control and serum metabolites in overweight/obese men: a randomised trial.

Research involving human participants, their data, or biological material

Policy information about studies with human participants or human data. See also policy information about sex, gender (identity/presentation), and sexual orientation and race, ethnicity and racism.

Reporting on sex and gender

This study is applicable to both sexes men and women. 7 cohorts were used. 2 cohorts only recruited men, 1 cohort primarily recruited women (96.6%) and the other 4 were a mix of men and women.

Reporting on race, ethnicity, or other socially relevant groupings

We didn't specifically study the effects of race or ethnicity on our results. This study was however conducted based on 7 cohorts. Each of the 7 cohorts recruited volunteers from a different different country. The countries were USA, UK, Denmark, Jordan, Germany, Qatar and Australia.

Population characteristics

For the Brigham cohort:
In total, 33 volunteers were recruited for the study aged between 18-75. The breakdown of the groups was as follows: Obese T2D: 8 participants (3 women, 5 men) with a BMI range of 30-45 and Hemoglobin A1c (HbA1c) levels between 5.7-9%. It is important to note that one participant was not adhering to their prescribed metformin regimen. Another individual had a BMI marginally below the initial target (29.8). Obese Pre-Diabetic: 3 participants (2 men, 1 woman), including one individual with a BMI slightly below the initial target (BMI 28.3). Obese Non-T2D: 11 participants (3 men, 8 women), with BMI ranges primarily within 30-45, including one individual with a BMI slightly below the initial target (29). Lean Pre-Diabetic: 1 participant (man) with a BMI of 19.6.
Lean Non-T2D: 10 participants (2 men, 8 women), all within the targeted BMI range for lean controls.

In brief for the other cohorts:

TwinsUK:.
2069 volunteers, 96.6% women, age at first sampling 32 to 73 years

Metformin increases endogenous glucose production in non-diabetic individuals and individuals with recent-onset type 2 diabetes
12 Diabetics (6 men,6 women) Age >50 BMI <40. 12 Healthy controls (6 men, 6 women) Age >50 BMI <40

A Metabolic Pattern in Healthy Subjects Given a Single Dose of Metformin: A Metabolomics Approach
26 healthy young male subjects from Jordan. Each participant, aged 18–50 years

Short-term variability of the human serum metabolome depending on nutritional and metabolic health status
10 Healthy Controls(7 women, 3 men) BMI: 19.9 - 27.9 , 10 prediabetic(4 women,6 men) BMI:28.3 - 41.9, 10 Type 2 diabetics (10 men) BMI 26.6 - 38.9

Metabolic changes of the blood metabolome after a date fruit challenge.
21 volunteers 13 females and 8 males. Volunteers with elevated blood glucose (>120 mg/dL) prior to the challenge was used as an exclusion criterion. BMI range 19.6 to 31.4

The effect of morning vs evening exercise training on glycaemic control and serum metabolites in overweight/obese men: a randomised trial.
24 overweight/obese sedentary men aged 30–45 years; BMI 27.0–35.0

Recruitment

For the Brigham cohort:
Volunteers for the study were recruited from the diabetes clinic and/or the weight management clinics at Brigham and Women's Hospital for the obese groups with or without Type 2 Diabetes (T2D). In addition, flyers placed in clinical and nonclinical areas within the hospital were used to recruit lean control subjects.

Inclusion Criteria
● Men and women between 18-75 years of age
● For lean controls: BMI of 18.5-24.9 for Caucasian and African American individuals, and 18.5-23.0 for Asian adults
● For obese cohorts, whether with or without prediabetes or diabetes, the inclusion criteria were a BMI of 30-45 and HbA1c levels of 5.7-9.5%
Individuals with diagnosed diabetes were predominantly on metformin, with or without insulin.
Exclusion Criteria
● Current smoker
● Pregnancy or planning pregnancy during the study period
● Serious uncontrolled cardiovascular, nervous system, pulmonary, renal, or gastrointestinal disease.
● Serologic evidence of current or past HIV, Hepatitis B, or Hepatitis C
● History of tuberculosis
● Liver disease with elevated hepatic enzymes

- Any of the following hematologic abnormalities on a blood test done in the last 12 months
  - White blood count <3,000/µL or >14,000/µL;
  - Lymphocyte count <500/µL;
  - Platelet count <150,000 /µL;
  - Hemoglobin <10 g/dL; or
  - Neutrophil count <2,000 cells/µL.
- Poor glycemic control (HbA1c >9.5% for the diabetes group)
- Major psychiatric illness
- Ongoing alcohol or drug abuse.

Recruitment for all other cohorts used are detailed in the original publications

Ethics oversight

Brigham cohort:
All volunteers provided written informed consent. This study was reviewed and approved by the Brigham and Women's Hospital Institutional Review Board (IRB 2020P002554 and 2019P001128).

Note that full information on the approval of the study protocol must also be provided in the manuscript.

Field-specific reporting

Please select the one below that is the best fit for your research. If you are not sure, read the appropriate sections before making your selection.

☒ Life sciences ☐ Behavioural & social sciences ☐ Ecological, evolutionary & environmental sciences

For a reference copy of the document with all sections, see nature.com/documents/nr-reporting-summary-flat.pdf

Life sciences study design

All studies must disclose on these points even when the disclosure is negative.

Sample size

The Brigham study was an exploratory study and we didn't use a statistical methodology to pre-determine sample sizes.

The sample sizes for all other studies were determined based on the specific research questions addressed in those original studies, which differ from our investigative focus on Lac-Phe during the reanalysis of their data. In all cases these untargeted metabolomic datasets had more than 500 metabolites measured. Our question was often more targeted - focusing on 1 or a small group of metabolites - thus typically need fewer samples.

The TwinsUK cohort utilized a significant subset of the cohort's participants for metabolomic analysis initially to explore the impact of low-frequency genetic variants on metabolite levels, necessitating a large dataset (6196 samples)

Study:Metformin increases endogenous glucose production in non-diabetic individuals and individuals with recent-onset type 2 diabetes

For the initial study, a sample size comprising 12 individuals with recent-onset Type 2 Diabetes (T2D) receiving metformin, another 12 T2D individuals receiving a placebo, and 12 healthy controls administered metformin was considered large enough to explore the primary outcome, which focused on whole-body glucose disposal and endogenous glucose production. Metabolomics analysis, serving as a secondary analysis, was conducted exclusively on the metformin-treated groups, omitting the placebo group from this part of the analysis.

A Metabolic Pattern in Healthy Subjects Given a Single Dose of Metformin: A Metabolomics Approach:
26 men underwent serial blood sampling. This sample size was considered sufficient to identify significant metabolic alterations following metformin administration. The study provided adequate statistical power to discern variations in more than 100 metabolites.

Short-term variability of the human serum metabolome depending on nutritional and metabolic health status:
The sample size was chosen: 30 volunteers sampled on 3 different days - before and after meals - to assess whether metabolite levels were stable over time. They were able to calculate and publish statistics on the stability of the metabolites across days.

Metabolic changes of the blood metabolome after a date fruit challenge.
A sample size of 21 volunteers was recruited, and serial blood draws were taken after three nutritional interventions . This sample size was considered sufficient to identify significant metabolic alterations following these different meals. The study provided adequate statistical power to discern variations in more than 50 metabolites.

The effect of morning vs evening exercise training on glycaemic control and serum metabolites in overweight/obese men: a randomised trial.
A sample size of 24 volunteers (3 groups: morning exercise n = 9 (8 completions); evening exercise n = 8; no exercise n = 8) was recruited. This sample size was considered sufficient to identify the effect of exercise training time on glycaemic control and serum metabolites. The study providing adequate statistical power to discern differences in glycaemic control and metabolites levels in the different groups.

A Metabolomic Signature of Glucagon Action in Healthy Individuals With Overweight/Obesity:
The sample size of thirty-three volunteers (Placebo, N = 10; GCG 12.5 ng/kg/min, N = 12; GCG 25 ng/kg/min, N = 11) was considered sufficient to identify significant energy expenditure alterations following Glucagon administration - no differences were found. Looking at differences in serum metabolites was a secondary analysis. The study provided adequate statistical power to discern individual serum metabolites and metabolite classes that changed in response to Glucagon

Data exclusions

In the TwinsUK dataset, we excluded samples that exhibited more than 175 missing metabolites, affecting 8 out of 6,196 samples (0.13%). Samples exceeding 175 missing metabolites represented clear outliers, suggesting potential issues with the sample quality or data acquisition. This threshold was selected based on the distribution of missing metabolites, which showed a smooth continuum excluding these outliers. This cutoff was determined post hoc, as it was informed by the observed distribution of missing data, rather than being pre-established.

Replication

We have 2 primary results.
All attempts at replication for both results were succesful

Specifically:
Lac-Phe increases with metformin treatment. 4 cohorts were used to demonstrate this.
Lac-Phe increases after feeding. 4 cohorts were used to demonstrate this.

Randomization

2 observational studies:
The Brigham cohort, no Randomization - For analysis, where appropriate we compared Obese T2D's vs. Obese Non T2D. We used further cohorts to validate our results.
The TwinsUK cohort,no Randomization - - To account for the lack of intervention/randomization we exploited the fact that we had 3 blood draws over time - with an average of 6.5 years between draws - per volunteer. The trajectory of Lac-Phe levels in volunteers was traced for those volunteers whose T2D and metformin status changed between subsequent samples, and we could compare this to volunteers whose had T2D and/or metformin status had not changed.

2 Interventional metformin studies

Study:Metformin increases endogenous glucose production in non-diabetic individuals and individuals with recent-onset type 2 diabetes : no Randomization for metabolomics.
The analysis of the metabolites was a secondary analysis of the study and as results randomization was not used. All subjects given metformin were sampled. However each volunteer was their own control because they were sampled before and after the metformin intervention.

A Metabolic Pattern in Healthy Subjects Given a Single Dose of Metformin: A Metabolomics Approach: no Randomization
Measurements were in the same individual. So metabolite levels were measured on the same day before and at timepoints after the administration of metformin. Each volunteer was their own control because they were sampled before and after the metformin intervention.

Short-term variability of the human serum metabolome depending on nutritional and metabolic health status: no Randomization
There was no randomization each volunteer was subject to a nutritional intervention on 3 separate days. However each volunteer was their own control because they were sampled before and after after the nutritional intervention.

Metabolic changes of the blood metabolome after a date fruit challenge: no Randomization
There was no randomization each volunteer was subject to each of three nutritional interventions. However each volunteer was their own control because they were sampled before and at timepoints after the interventions.

One study had randomization for an exercise intervention : The effect of morning vs evening exercise training on glycaemic control and serum metabolites in overweight/obese men: a randomized trial
Upon analyzing the effects of the randomized exercise intervention, we found that it did not exert a significant influence on the outcomes of our study. This analysis ensured that the intervention's impact was thoroughly evaluated. However, because our study primarily focused on the effects of sampling time relative to meals, we chose not to include detailed data regarding this exercise intervention in our findings.
Volunteers were measured before breakfast and after dinner, so volunteers could act as their own controls.

Metabolomic Signature of Glucagon Action in Healthy Individuals With Overweight/Obesity:Randomization
The were three groups a placebo group a low dose Glucagon group and a high dose glucagon groups. These groups were assigned by randomisation. The author provide details in another publication based on this cohort - which related to their primary endpoint which was energy expenditure:Prolonged Glucagon Infusion Does Not Affect Energy
Expenditure in Individuals with Overweight/Obesity:
A Randomized Trial:
"Participants were stratified into groups
on the basis of sex and BMI (≥36, <36 kg/m2
) prior to randomization to
one of the three groups using a pseudorandom number generator in JMP
(version 13.0; SAS Institute Inc., Cary, North Carolina) "

Blinding

2 of the studies the Brigham study and the TwinsUK cohort were observational study. Please see the Randomization section for how we controlled for this

Study:Metformin increases endogenous glucose production in non-diabetic individuals and individuals with recent-onset type 2 diabetes :
The analysis of the metabolites was a secondary analyis of the study and as results blinding was not used. All subjects given metformin were sampled. However each volunteer was their own control because they were sampled before and after the metformin intervention.

A Metabolic Pattern in Healthy Subjects Given a Single Dose of Metformin: A Metabolomics Approach:
All volunteers we admininsted the intevention (taking a single dose of metformin). However each volunteer was their own control because they were sampled before and at multiple time points after the metformin intervention.

Short-term variability of the human serum metabolome depending on nutritional and metabolic health status:

There was no blinding each volunteer was subject to a nutritional intervention on 3 separate days. However each volunteer was their own control because they were sampled before and after after the nutritional intervention.

Metabolic changes of the blood metabolome after a date fruit challenge.
There was no blinding each volunteer was subject to each of three nutritional interventions. However each volunteer was their own control because they were sampled before and at timepoints after the interventions.

The effect of morning vs evening exercise training on glycaemic control and serum metabolites in overweight/obese men: a randomised trial. Blinding would not have made sense in this cohort because the different groups performed either no exercise, exercise in the morning and exercise in the evening. It would be impractical to hide this informations from the participants or the researchers supervising the study.

A Metabolomic Signature of Glucagon Action in Healthy Individuals With Overweight/Obesity:
The were three groups a placebo group a low dose Glucagon group and a high dose glucagon groups. Blinding was conducted.
The author provide details in another publication based on this cohort - which related to their primary endpoint which was energy expenditure:Prolonged Glucagon Infusion Does Not Affect Energy
Expenditure in Individuals with Overweight/Obesity:
A Randomized Trial: "All researchers and participants were blinded to assignment
except the pharmacist issuing the glucagon"

Reporting for specific materials, systems and methods

We require information from authors about some types of materials, experimental systems and methods used in many studies. Here, indicate whether each material, system or method listed is relevant to your study. If you are not sure if a list item applies to your research, read the appropriate section before selecting a response.

Materials & experimental systems

n/a	Involved in the study
☒	☐ Antibodies
☒	☐ Eukaryotic cell lines
☒	☐ Palaeontology and archaeology
☒	☐ Animals and other organisms
☒	☐ Clinical data
☒	☐ Dual use research of concern
☒	☐ Plants

Methods

n/a	Involved in the study
☒	☐ ChIP-seq
☒	☐ Flow cytometry
☒	☐ MRI-based neuroimaging

Plants

Seed stocks	*Report on the source of all seed stocks or other plant material used. If applicable, state the seed stock centre and catalogue number. If plant specimens were collected from the field, describe the collection location, date and sampling procedures.*
Novel plant genotypes	*Describe the methods by which all novel plant genotypes were produced. This includes those generated by transgenic approaches, gene editing, chemical/radiation-based mutagenesis and hybridization. For transgenic lines, describe the transformation method, the number of independent lines analyzed and the generation upon which experiments were performed. For gene-edited lines, describe the editor used, the endogenous sequence targeted for editing, the targeting guide RNA sequence (if applicable) and how the editor was applied.*
Authentication	*Describe any authentication procedures for each seed stock used or novel genotype generated. Describe any experiments used to assess the effect of a mutation and, where applicable, how potential secondary effects (e.g. second site T-DNA insertions, mosiacism, off-target gene editing) were examined.*
